# Supplementary material for: Knowledge and application of sonographic scoring models for ovarian cancer management among gynecologists in Saudi Arabia: a cross-sectional study
Source: PeerJ. 2025 Jul 29;13:e19746. doi: 10.7717/peerj.19746 (PMC12315828; doi:10.7717/peerj.19746)
Supplement: Supplemental Information 2 [file peerj-13-19746-s002.docx]

**Assessing Knowledge and application of widely used sonographic scoring models among Gynaecologist in Saudi Arabia to diagnose ovarian masses and the impact of them in decision to intervene in patients**

Dear DR/RDA

Kindly, answer the questions within the attached questionnaire. This questionnaire is a part of a study to explore the knowledge about and application of the widely used scoring models to diagnose ovarian masses and the contribution of them in the decision making for surgery. You can agree or disagree to answer the questionnaire with your free will.

I appreciate your effort in answering the questions in the questionnaire, knowing that the given information will be used for the purpose of scientific study only and will be treated confidentially.

Thank you for your cooperation.

Section (A): Demographic Characteristics

1) Gender: Male	Female

What is the region in which you work?

2.

3.

4.

5.

6.

7.

8.

9.

10.

11.

12.

13.

3) What is your position:

-Specialist

-Gynaecology Consultant

-Gynae oncology consultant

4) Experience in years

-2-5 years

-6-10 years

-11-15 years

-More than 15 years

5) Type of current hospital:

-Secondary hospital (150 beds or more)

-Specialized hospital (tertiary)

-Teaching and Research Hospital

6) How many patients you see in your gynaecology clinic per week?

-less than 10

-10-20

-21-30

-31-40

Section (B): Knowledge and Experience

The statements below indicate your level of agreement or disagreement. There are no correct or wrong answers. Please use the scale indicated below

Scale:

1=Strongly disagree	2=Disagree	3=Neutral	4=Agree	5=Strongly Agree

7) I have received training in Gynaecology ultrasound scanning

Strongly agree

Agree

Neutral

Disagree

Strongly disagree

8) If you are not the one scanning the patients, your ultrasound department have an experienced reliable sonographers

Strongly agree

Agree

Neutral

Disagree

Strongly disagree

I scan the patient myself

9) There are several different types of sonographic scoring models for diagnosing benign from malignant in adnexal masses

Strongly agree

Agree

Neutral

Disagree

Strongly disagree

10) I am aware of the risk of malignancy index (RMI) to differentiate benign from malignant ovarian masses (I read about it and know how to calculate it)

Strongly agree

Agree

Neutral

Disagree

Strongly disagree

11) I am aware of the newest model (ADNEX) developed by the IOTA group

Strongly agree

Agree

Neutral

Disagree

Strongly disagree

12) The ADNEX model needs to be validated nationally before it can be used in clinical setting

Strongly agree

Agree

Neutral

Disagree

Strongly disagree

Not applicable

13) I am always updated with the recent international guidelines for patient management with ovarian masses

Strongly agree

Agree

Neutral

Disagree

Strongly disagree

Section (C): Factors affecting decision making for surgery

14) Which of these factors were considered in decision making for surgery (please tick all boxes that apply) (patient examination, symptoms and history is outside the scope of this study)

-Overall ultrasound morphology of the ovary

-Ovarian diameter and size

-Attention to any of the following specific features e.g. irregular wall, number of cysts, septation, solid areas, papillation or echogenicity

-Presence of abnormal morphology on both sides

-Presence of doppler signal in abnormal area

-Presence of ascites

-Results of tumour marker CA-125

-Other tumour marker

-Risk of malignancy index (RMI)

-ADNEX model

-MRI magnetic resonance imaging

-CT computed tomography

-Other considerations: general medical health and suitability for surgery

-Patient wishes

15) Did you calculate the risk of malignancy index (RMI) for your patients, where [RMI= U x M x CA125]

Yes

No

Some of them

16) If you answered No to previous question, indicate the reason please______________

17) If you answered Yes to question 15, which version of RMI was used:

-RMI I [U=0 if no abnormality detected, U=1 if one abnormality seen, and U=3 if >1 --abnormality seen, for postmenopausal M=3]

-RMI II [U=1 if no or 1 abnormality seen, U=4 if >2 abnormality detected, for -------- ----_postmenopausal M=4]

-Other version (RMI IV)

-Not applicable

18) Did the total score of RMI affect the decision for surgical intervention

Yes

No

Some of them

Not applicable

19) Please explain the reason for your answer to previous question 18_________________

20) Do you calculate ADNEX model for your patient

Yes

No

Some of them

21) D0 you use other morphologic scoring system to diagnose ovarian masses?

Yes

No

Sometimes

Rarely

22) Please specify the name of the scoring system you are using and the reason for using it_________________________________________________
